# Right Colectomy with Complete Mesocolic Excision and Intracorporeal Anastomosis: A Monocentric, Single-Surgeon Comparison of Dexter, DaVinci and Laparoscopic Approaches

**DOI:** 10.3390/life15071122

**Published:** 2025-07-17

**Authors:** Julius Pochhammer, Frederike Franke, Matthias Martin, Jan Henrik Beckmann, Daniar Osmonov, Ibrahim Alkatout, Thomas Becker

**Affiliations:** 1 Department of General, Abdominal, Thoracic, Transplantation and Pediatric Surgery, University Hospital Schleswig-Holstein, Campus Kiel, Arnold-Heller-Straße 3, 24105 Kiel, Germany; julius.pochhammer@uksh.de (J.P.);; 2 Department of Urology, University Hospital Schleswig-Holstein, Campus Lübeck, Ratzeburger Allee 160, 23538 Lübeck, Germany; 3 Department of Gynecology and Obstetrics, University Hospital Schleswig-Holstein, Campus Kiel, Arnold-Heller-Straße 3, 24105 Kiel, Germany

**Keywords:** CME, hemicolectomy, colorectal cancer, robotic surgery, minimally invasive surgery

## Abstract

(1) Minimally invasive techniques are standard in colorectal surgery, though complete mesocolic excision (CME) with central lymphadenectomy remains technically demanding. Robotic systems may address these challenges. While the DaVinci system is well established, the modular Dexter system allows rapid switching between laparoscopy and robotics. (2) This prospective single-surgeon study compared right hemicolectomy with CME and intracorporeal anastomosis using Dexter, DaVinci, and conventional laparoscopy in 75 patients (25 per group) at a German high-volume center. Outcomes assessed included operative time, complications, lymph node yield, and CME quality. (3) Mean operative time was longest with DaVinci (190.5 min) versus Dexter (164.8 min) and laparoscopy (152.6 min). Intracorporeal anastomosis was more frequent in robotic groups. No significant differences were found in lymph node yield, CME quality, postoperative complications, length of stay, or survival. (4) The ability to convert briefly to laparoscopy during Dexter procedures helped manage challenging steps, especially during the learning curve. The results suggest that Dexter is a safe, feasible alternative to established robotic and laparoscopic techniques, with the added benefits of flexibility and integration into existing workflows.

## 1. Introduction

Following the gradual development of minimally invasive surgery over the last century, since the 1990s, it has become a successful model and the standard approach for many procedures in recent decades [1,2].

The advantages, which include reduced postoperative pain, reduced blood loss, and fewer postoperative complications, as well as faster recovery, are well known. Based on several multicentric trials, a minimally invasive approach is recommended for right hemicolectomy, for example [3,4,5], and has become the standard approach nowadays, representing 90.5–97.1% of cases in several countries [6,7,8]. However, laparoscopy faces several technical limitations inherent to the technique. Central lymphadenectomy and complete mesocolic excision (CME), for example, can be technically challenging as laparoscopic instruments cannot be angled. This can lead to inadequate radicality and is connected to poorer patients survival [9]. There is further restriction when creating an intracorporeal anastomosis, which is technically demanding laparoscopically. Therefore, it is often not performed but offers advantages such as a shorter hospital stay and earlier solid food intake [6,10,11]. In addition, the risk of hernia is lower after a suprapubic Pfannenstiel incision for specimen retrieval than for periumbilical incision to perform extracorporeal anastomosis [12].

The development of robotic technologies has revolutionized the field of minimally invasive surgery, offering precision, enhanced dexterity with articulating instrumentation, three-dimensional visualization, and improved ergonomics for the surgeon [13]. The non-inferiority of robotic assistance compared to laparoscopic procedures has already been demonstrated for various entities. There is still no high-level evidence for hemicolectomy, though. However, a meta-analysis of various monocentric comparative studies showed a better harvesting of lymph nodes, lower conversion rate to open surgery, a shorter time to first passage of stool, and a shorter length of stay compared to laparoscopy [14,15]. This is accompanied by a longer operative time [16].

After 20 years of DaVinci being the only robotic system on the market, new robotic systems have recently entered the market, demonstrating their feasibility and safety in a variety of procedures [17]. However, if these systems are to survive on the market, they must demonstrate good practicability in comparison with existing systems.

The Dexter robotic surgery system (Distalmotion SA, Epalinges, Switzerland) received CE marking in 2020 for use in gynecological, urological and general surgical procedures. It represents a modular and easily accessible approach to robotic surgery. The system can be integrated seamlessly into the established laparoscopic workflow, reducing barriers to the adoption of a robotic program. Surgeons working at a sterile console enable fast switching between laparoscopy and robotics in up to 15–30 s [18]. The objective of this novel approach is to address the principal limitations of robotic-assisted surgery and facilitate its widespread adoption, in particular in high-volume and routine procedures of moderate complexity. The feasibility and safety of the Dexter system have previously been documented in urology [19,20], gynecology [21,22] and general visceral surgery [18,23,24,25].

This study aims to investigate and compare the practicability of two different robotic systems and conventional laparoscopy in right hemicolectomy with CME and intracorporeal anastomosis. For this purpose, 25 consecutive procedures performed by one surgeon in a single center using identical structural situations with each approach (Davinci Xi, Dexter and laparoscopy) were retrospectively compared.

## 2. Materials and Methods

### 2.1. Study Design and Patients

This prospective cohort study included 25 consecutive patients in each group who underwent minimally invasive right hemicolectomy for oncological reasons with intended CME between April 2021 and April 2024 by the same surgeon. All procedures for which a right hemicolectomy with CME was intended and performed by the author were included in this study. CME was performed with D3-Lymphadenectomy using the “open-book-model” technique [26]. In the Dexter (DXRC) group, we used the Dexter system; in the DaVinci (DVRC) group, a DaVinci Xi system (Intuitive, Sunnyvale, CA, USA); and in the laparoscopic (LRC) group, an Olympus Visera III laparoscopic System (Olympus Europa SE, Hamburg, Germany). Allocation to the procedures took place in the first period of the study between 2021 and 2022 in the DVRC group, and if the system was available, alternatively in the LC group. Between January 2023 and November 2024, the Dexter system was also available, so when available, patients were included in the DXRC group, or alternatively in the DVRC or LC group. All procedures were performed for colon cancer in a German academic high-volume center. At the beginning of this study, the surgeon had experience in 36 laparoscopic CME procedures, as well as 29 and 76 different robotic-assisted surgical procedures in DXRC and DVRC groups, respectively, but no experience in robotic CME procedures.

All procedures were conducted in accordance with the ethical standards of the respective local ethic committees and with the Helsinki Declaration of 1964 and later versions. The faculties ethics committee approved this study beforehand and written informed consent was obtained prior to providing the treatment.

### 2.2. Variables and Definitions

The baseline patient characteristics collected included sex, age, pre-existing diseases, American Society of Anesthesiologists (ASA) score, and Body Mass Index (BMI). Operative variables collected included operative time (time between the first incision and final skin closure) and docking time (for DXRC and DVRC groups; time between first approach of the robotic system and first instrument movement of the console). Furthermore, conversion to open surgery and method of anastomosis, as well es quality of resection and lymph node harvest, were analyzed. The proportion of laparoscopy was calculated for the DXRC group as the ratio of minutes operated by laparoscopic approach divided by the total minutes between docking and end of anastomosis suture. Postoperative outcomes were occurrence of postoperative complications and mortality, type of complication, and length of hospital stay. Complications were graded according to the Clavien–Dindo scale [27]. An anastomotic leak was defined as an endoscopically or surgically proven defect of the intestinal wall at the anastomotic site. Surgical site infections (SSIs) were diagnosed according to the criteria of the Centers for Disease Control and Prevention [28].

### 2.3. Surgical Technique

Mechanical bowel preparation, combined with oral antibiotic application of neomycin and metronidazole, was intended in all patients without symptomatic stenosis the day before surgery. Preoperatively, patients received intravenous administration of 2 g cefuroxime and 500 mg metronidazole; in case of cephalosporin allergy, 500 mg ciprofloxacin was administered instead. Administration before skin incision was verified by checklist and was not continued after surgery.

**Figure 1:**
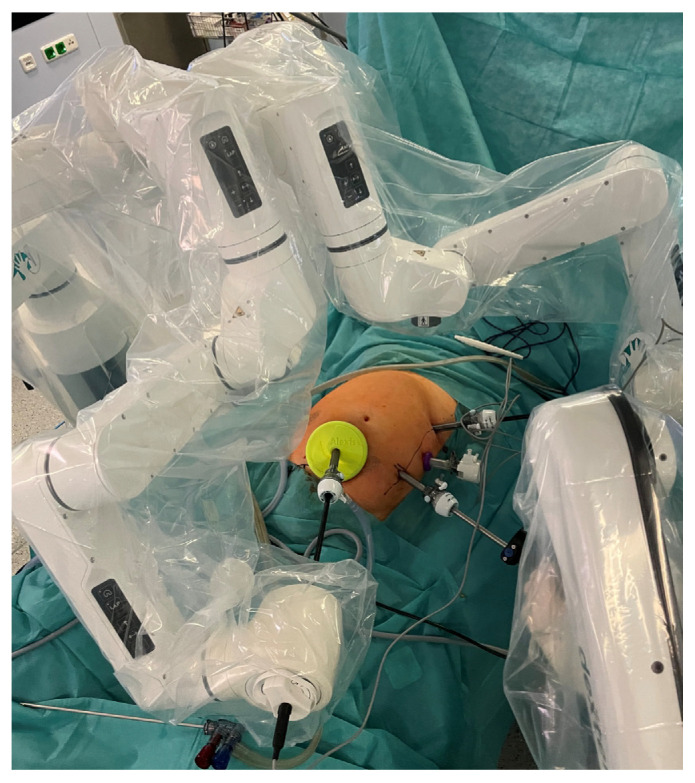
Trocar positions in Dexter procedure.

**Figure 2:**
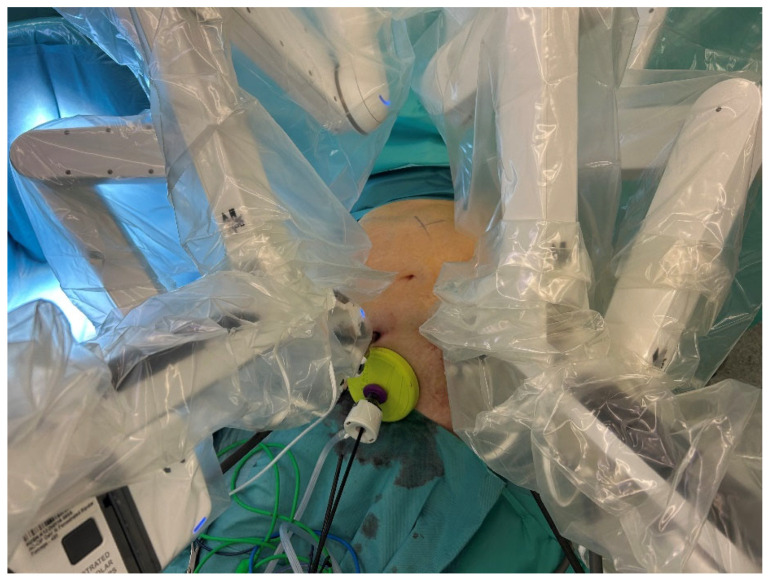
Trocar positions in DaVinci procedure.

In all three groups, patients were positioned with their arms along their body and their legs closed. In the DXRC and DVRC groups, the operating table was slightly tilted to the left (5°) in the anti-Trendelenburg position (5°). For LRC, the procedure was started in Trendelenburg position (20–25°) and right-tilted (10°), and then switched to the position as described above. The positions of trocars are shown in Figure 1 and Figure 2. All procedures started via mini-laparotomy and usage of a Alexisport (Size S; Applied Medical, Amersfoort, The Netherlands). In DXRC and DVRC groups, 3 and 4 robotic ports with 1 assistant port were used, respectively. In LRC 4-5 ports were used. In the robotic groups, the surgery was started with a subileal approach; in LRC, a retroduodenal approach was used to prepare the right mesocolon. All subsequent steps were identical across all groups, and were performed as previously described [26,29]. For the intracorporeal anastomosis, a Signia Stapling system (Medtronic plc, Dublin, Ireland) was used. The remaining opening was closed continuously in a single layer with a barbed suture (Stratafix; Ethicon Inc., Cincinnati, OH, USA). A closed-system intra-abdominal drainage was used according to the surgeon’s instructions. Risk-adjusted low-molecular-weight heparin was administered postoperatively for thromboembolic prophylaxis.

### 2.4. Statistical Analysis

All study parameters were prospectively recorded in our colorectal database and analyzed using the JMP 17.2 software (SAS Institute Inc., Cary, NC, USA). Differences in qualitative data were analyzed using the chi-square test; quantitative data were analyzed using the Wilcoxon–Mann–Whitney U test or one-factorial ANOVA with normal distribution. A *p*-value  <  0.05 was considered statistically significant. To compare the duration of surgery in the different groups, a paired-sample Student’s *t*-test was carried out. A multivariate analysis was performed for the duration of surgery, including all relevant factors from Table 1 and Table 2, and consisted of a multiple regression analysis with backward elimination. The procedure was repeated until no explanatory variable remained that could be removed without significantly worsening the prediction of the endpoint.

## 3. Results

We operated on 75 patients during this period. A comparison of demographic data is shown in Table 1. All variables, i.e., sex, age, BMI, pre-existing diseases, history of abdominal surgery, and tumor staging, showed no significant difference.

**Table 1:** Baseline data.

	DXRC	DVRC	LRC	*p*-Value
N	25	25	25	
Age	67.1 ± 13.3	67.0 ± 14.7	68.1 ± 11.5	0.95
Female gender	13 (52.0)	9 (36.0)	13 (52.0)	0.42
BMI	26.3 ± 4.3	27.1 ± 4.2	25.3 ± 4.2	0.34
Pre-existing diseases				
Cardiovascular	8 (32)	9 (36)	7 (28)	0.83
Arterial hypertension	11 (44)	6 (24)	14 (56)	0.07
Pulmonary	0	3 (12)	2 (8)	0.22
Metabolic	4 (16)	5 (20)	5 (20)	0.92
Renal	1 (4)	0	1 (4)	0.60
ASA ≥ 3	6 (24.0)	7 (28.0)	3 (12.0)	0.36
Previous abdominal surgery	8 (32)	11 (44)	7 (28)	0.47
Tumor stage (UICC)				
HGIEN	3 (12)	2 (8)	1 (4)	
I	5 (20)	4 (16)	4 (16)	
II	8 (32)	9 (36)	12 (48)	
III	5 (20)	7 (28)	4 (16)	
IV	4 (16)	3 (12)	4 (16)	0.93
MMR deficiency	10 (40)	8 (32)	6 (24)	0.46

Data are given as n (%) or mean ± SD. BMI: Body Mass Index; ASA: American Society of Anesthesiologists Physical Status System; MMR: Mismatch repair; UICC: Union Internationale Contre le Cancer; HGIEN: High-grade intraepithelial neoplasia.

**Figure 3:**
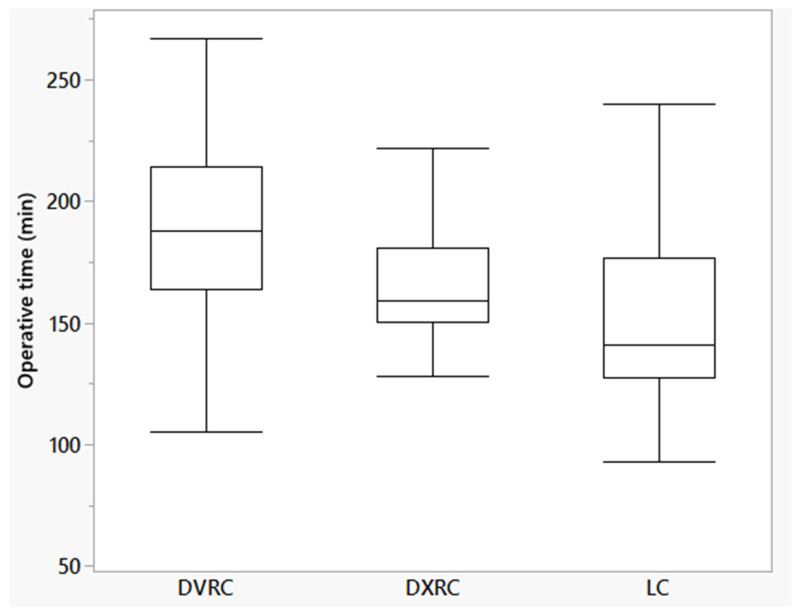
Box-plot of operative time of all procedures. DVRC: DaVinci group; DXRC: Dexter group; LC: Laparoscopic group.

The perioperative outcomes are listed in Table 2. Extracorporeal anastomosis was performed significantly more often in LRC group than in robotic procedures. In the latter, the anastomoses were completely sutured intracorporeally in 20% and 24% of cases, respectively. The operating time was significantly longer in the DVCR group than in DXCR and LCR (190.5, 164.8, and 152.6 min, respectively; *p* < 0.01). No significant difference was found between DXCR and LCR (Figure 3). In the multivariate analysis, BMI, fulfillment of CME, type of surgical approach, and duration of docking were included in the final model. The *p*-values were <0.01, <0.01, <0.05, and 0.19, respectively, indicating that MI, performance of CME, and type of surgical approach (group membership) were independent influencing factors.

Since the transversus abdominis plane block was introduced over the course of the study, it was used more frequently in the DXCR group. The same applies to the stapler magazines used. Due to some intraluminal bleeding events from the anastomosis area, lower staple heights were used from this time onwards, which is reflected in the different applications in the groups (*p* < 0.01). A change in the standard is also evident in the use of drains, which were no longer used as standard in the second phase of the study due to a lack of evidence. They were therefore inserted less frequently in the DXCR group (*p* < 0.01).

**Figure 4:**
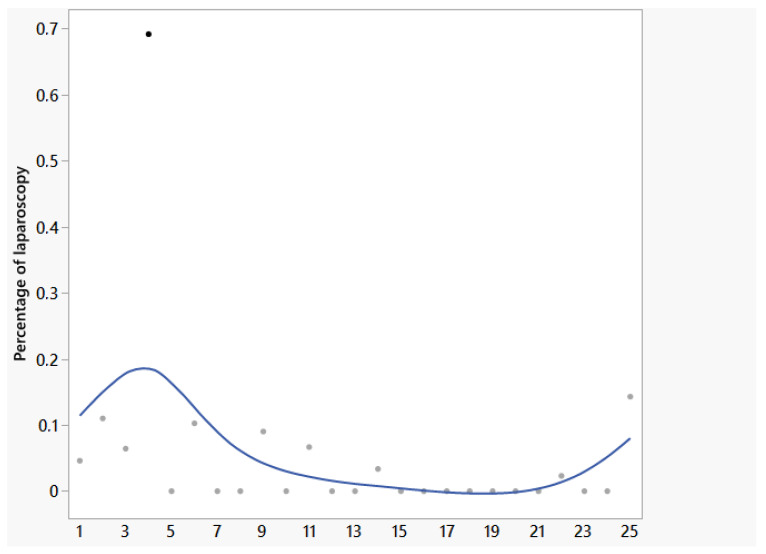
Percentage of laparoscopic surgery in Dexter robotic procedures over time.

The planned CME was not carried out in all procedures, whether due to extensive distant metastases or poor intraoperative patient condition (7 cases). If a CME was performed, a CME quality type 0 according to Benz (determined by pathology) was achieved in 95.6% of cases [30]. One conversion occurred in the laparoscopic group due to a feared infiltration of the anterior renal fascia, which was not pathologically confirmed but could not be resolved by minimally invasive surgery. For DXRC procedures, the use of conventional laparoscopy was decreased, with a median of 0% and a range of 0% to 69% (Figure 4). The median numbers of harvested lymph nodes were 22, 28, and 26 in DXCR, DVCR, and LCR groups, respectively, and showed no significant difference. No residual cancer cells at the resection margin were found in any of the groups.

**Table 2:** Intraoperative data.

	DXRC	DVRC	LRC	*p*-Value
Type of anastomosis				
Extracorporeal hand-sewn	0	1 (4)	11 (44)	
Intracorporeal stapled	20 (80)	18 (72)	14 (56)	
Intracorporeal hand-sewn	5 (20)	6 (24)	0	<0.01
Operative time (min [95% confidence interval])	164.8 (151.7–178.0)	190.5 (177.3–203.7)	152.6 (139.4–165-7)	<0.01
Initial docking (min)	5.6 ± 2.5	9.6 ± 3.4	n/a	<0.01
TAP-block	13 (52.0)	7 (28.0)	5 (20.0)	0.04
Height of staples (mm)				
1.5–2.5 (blue)	22 (88)	11 (44)	18 (72)	
3.0–4.0 (purple)	3 (12)	10 (40)	7 (28)	
1.8–3.0 (gold)	0	1 (4)	0	
4.3 (green)	0	3 (12)	0	0.02
Conversion to open	0	0	1 (4)	0.88
Percentage of laparoscopy (%)	0 (0–69)	0	100	-
Harvested lymph nodes	22 (10–60)	28 (12–46)	26 (10–47)	0.18
CME fulfilled	24 (96)	22 (88)	22 (88)	0.53
Pathological classification Benz-type 0 if CME fulfilled	24 (100)	20 (90.9)	21 (95.5)	0.32
Intra-abdominal drainage	1 (4)	16 (64)	6 (24)	<0.01

Data are given as n (%), median (min–max) or mean ± SD. TAP-block: Transverse abdominis plane block; n/a: not available; CME: Complete mesocolic excision.

**Figure 5:**
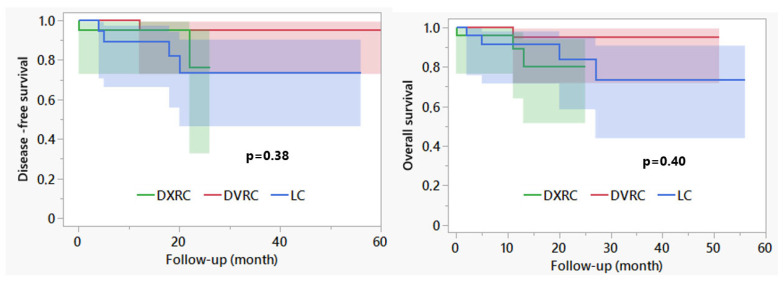
Overall survival in different groups. Disease-free survival and overall survival given with 95% confidence interval. DVRC: DaVinci group; DXRC: Dexter group; LC: Laparoscopic group.

The overall postoperative outcomes are reported in Table 3. The length of stay was not significantly different between the groups; the median was 4 days in the robotic groups, and 5 days after LRC. The complications were comparable between the three groups. One patient with serious pre-existing cardiac disease (aortic valve stenosis) but stenosing tumor disease died of irreversible cardiac arrest 7 days after surgery. The median follow-up of the entire cohort was 17 months (1–56); the disease-free and overall survival rates are shown in Figure 5 and did not differ significantly. The local recurrence rates were 4%, 4% and 12% for DXRC, DVRX and LC groups, respectively (*p* = 0.42).

## 4. Discussion

This study demonstrated that clinical results after hemicolectomy with CME and intended intracorporeal anastomosis are comparable across the Dexter system, DaVinci system, and conventional laparoscopy. Meta-analyses have now repeatedly proven the advantage of CME in oncological right hemicolectomy; the feasibility of robotic execution has also been proven [31]. Therefore, in this study, we tried to emphasize the implementation with different robotic systems. A monocentric, single-surgeon structure was chosen to enable comparability despite the small number of cases.

A comprehensive literature review by Ahmad et al. showed that robotic-assisted surgery had greater advantages in confined spaces, such as in rectal cancer surgery, while it had no obvious advantage in colorectal cancer surgery [32]. However, a systematic review and meta-analysis suggested that robotic surgery represented lower conversion rates, faster recovery of bowel function, shorter length of hospital stay, and lower complication rate [33]. It was, however, undeniable that a longer operative time and higher costs were observed in robotic surgery compared to those in laparoscopic [34]. As the costs for the systems and consumables are currently dependent on negotiations and there are significant differences between institutions, it is not possible to make a meaningful comparison of the cost situation here. However, it can be assumed that there are significant additional costs for robotic procedures compared to laparoscopic procedures and that DVRC incurs higher additional costs than DXRC.

The available data comparing laparoscopic and robotic interventions are essentially retrospective in nature. Nevertheless, matched studies consistently show longer operating times for the robotic approach, with a shorter hospital stay. In addition, the lymph node yield is generally higher after robotic procedures. This was also confirmed in recently published meta-analyses [35]. Long-term survival appears to be comparable for both procedures [36]. However, the need for high-quality evidence is repeatedly emphasized.

Our study is the first to investigate the novel Dexter robotic system in that entity, which enables a simpler transition between laparoscopic and robotic procedures due to its modular design. It shows that the longer operating times shown for robotic procedures can be significantly reduced to the level of the laparoscopic approach. The shorter operating time seems to be possible not only due to shorter docking times, but also due to the modular, simpler set-up [25]. During the learning curve, subileal access can be very time-consuming at the beginning of the procedure in obese patients, as repositioning in the Trendelenburg position is not possible without undocking. In this case, the temporary switch to a laparoscopic approach with DXRC could speed up the procedure somewhat. The subileal approach goes hand in hand with the suprapubic trocar position; whether a retroduodenal approach, as described by Benz et al., shortens operating times should be examined [29]. Nevertheless, the length of hospital stay appears to be comparable to the purely robotic approach, and the lymph node yield is not different either.

The reported 25 Dexter procedures included the learning curve of the surgeon with the Dexter system, who was otherwise robotically experienced. The ability to briefly switch between modalities makes it easier during that learning phase of navigating more difficult parts of the procedure [37]. On the other hand, we can see that the percentage of laparoscopic steps decreased over time, with the median percentage of laparoscopy in DXRC cases being zero. The increased proportion of laparoscopic procedures at the beginning of the study could be due to increasing experience with port placement (Figure 5). We found that this was essential to reach the required areas of the abdomen. The increased value in the last procedure performed was due to the need for mobilization of the transverse colon in obese patients.

The use of the Dexter robotic system could be advantageous for procedures such as right hemicolectomy with CME. The procedure is normally limited to two abdominal quadrants, which reduces the difficulty of reaching more than two quadrants without the new docking of Dexter. On the other hand, this procedure requires less static holding force than, for example, deep anterior rectal resection. Nevertheless, the robotic support for fine preparation of the central lymphadenectomy is a considerable advantage that users might prefer.

The anastomosis technique varied over the course of the study. In the laparoscopic group, extracorporeal sutured anastomoses were performed at the beginning, as this corresponded to our standard. With the introduction of robotic right hemicolectomy, laparoscopic anastomoses were also performed intracorporeally, although suturing the opening remaining after stapling is more complex due to the lack of intra-abdominal angulation. However, there is now some evidence in favor of the intracorporeal procedure [10]. With increasing robotic experience, completely sutured side-to-side anastomoses were also performed intracorporeally with double-armed, barbed suture, as this hardly increases the time required and requires fewer staplers. In our small group, there were no disadvantages in terms of safety, but further evaluative comparisons are necessary. For intracorporeally stapled anastomoses, we used laparoscopic stackers, as these are less expensive than the robotic version.

## 5. Limitations

The main limitation of this study lies in the monocentric, retrospective approach. The experience shown also implies the surgeon’s learning curve for procedures with both robotic platforms. As the procedures with Dexter only began in 2022, this may already represent progress in the overall robotic learning curve. In addition, the DXRC was closely monitored by the manufacturer, with recommendations for improving the trocar positions, for example. However, extensive external experience already exists for DVRC, which can be used as a guide. The intraoperative approach is a strictly standardized procedure that has already been published as an open-book model [26]. This allowed us to adapt the procedural steps quickly and to establish comparability and transferability. There are differences in the groups concerning the use of drainage, for example. In the course of the study, guidelines advised against their routine use. Implementation could influence the length of stay in favor of later DXRC procedures, but these do not differ significantly [38]. The survival times of the groups do not differ significantly, but it must be noted that the follow-up times are short and differ between the groups, as the DXRC procedures, for example, tended to take place in the second half of the study (Figure 5). In addition, this study is not powered for tumor-specific endpoints, as the aim of this study was merely to test the usability of the Dexter system in everyday life.

Randomized studies are required to obtain better evidence. However, due to the high investment costs, it is unlikely that both systems will be available at a facility in addition to laparoscopy. Therefore, demonstrating feasibility and good outcomes for one entity may allow for selection of a system based on the facility’s procedure spectrum.

## 6. Conclusions

The Dexter system enables good, oncologically accurate dissection in right hemicolectomy with CME and central lymphadenectomy. Possibly due to the compact design and short docking phases, the duration of the procedures in this cohort were comparable to laparoscopic and possibly shorter than in DaVinci-assisted procedures. The uncomplicated change between laparoscopy and robotics possibly makes it easier to overcome difficult procedure sections during the learning curve. Even as a circumscribed, monocentric, single-surgeon study, our results suggest that application of both Dexter and DaVinci robotic platforms to right colectomy with CME and D3-lymphadenctomy is related to comparable safety profiles. Although DaVinci remains the most widely adopted platform in clinical practice, this study underscores the potential role of the Dexter robotic system to provide an effective solution for these procedures.

## Figures and Tables

**Table 3:** Postoperative results.

	DXRC	DVRC	LRC	*p*-Value
Length of stay	4 (3–10)	4 (3–48)	5 (2–35)	0.14
Complications within 30 days				
Acute pancreatitis (CD II)	0	0	1 (4)	
Intraluminal bleeding	0	1 (4)	0	
Colonoscopy (IIIa)			0	
Intraabdominal bleeding	0	1 (4)		
Re-laparoscopy (CD IIIb)				
Anastomotic leakage (CD IIIb)	1 (4)	1 (4)	2 (8)	
Mortality due to cardiac arrest (CD V)	1 (4)	0	0	
Surgical site infections	1 (4)	0	2 (8)	0.35
Follow-up	8 (1–24)	17 (1–51)	19 (1–56)	<0.01

Data are given as n (%) or median (min–max); CD: Clavien–Dindo Classification.

## Data Availability

The original contributions presented in this study are included in the article. Further inquiries can be directed to the corresponding author.

## Abbreviations

The following abbreviations are used in this manuscript:

ASA	American Society of Anesthesiologists Physical Status System
BMI	Body Mass Index
CD	Clavien–Dindo Classification
CME	Complete mesocolic excision
HGIEN	High-grade intraepithelial neoplasia
MMR	Mismatch repair
UICC	Union Internationale Contre le Cancer
